# Evolving Diagnostic and Management Advances in Coronary Heart Disease

**DOI:** 10.3390/life13040951

**Published:** 2023-04-05

**Authors:** Matheus de Oliveira Laterza Ribeiro, Vinícius Machado Correia, Lucas Lentini Herling de Oliveira, Paulo Rogério Soares, Thiago Luis Scudeler

**Affiliations:** Instituto do Coração (InCor), Hospital das Clínicas da Faculdade de Medicina da Universidade de São Paulo, São Paulo 05403-010, Brazil

**Keywords:** coronary artery disease, coronary intervention, atherosclerosis

## Abstract

Despite considerable improvement in diagnostic modalities and therapeutic options over the last few decades, the global burden of ischemic heart disease is steadily rising, remaining a major cause of death worldwide. Thus, new strategies are needed to lessen cardiovascular events. Researchers in different areas such as biotechnology and tissue engineering have developed novel therapeutic strategies such as stem cells, nanotechnology, and robotic surgery, among others (3D printing and drugs). In addition, advances in bioengineering have led to the emergence of new diagnostic and prognostic techniques, such as quantitative flow ratio (QFR), and biomarkers for atherosclerosis. In this review, we explore novel diagnostic invasive and noninvasive modalities that allow a more detailed characterization of coronary disease. We delve into new technological revascularization procedures and pharmacological agents that target several residual cardiovascular risks, including inflammatory, thrombotic, and metabolic pathways.

## 1. Introduction

Coronary artery disease (CAD) is the most common type of heart disease, killing approximately 380,000 people in 2020 [1]. Treatment of CAD includes lifestyle changes, risk factor management, drugs, and invasive procedures (percutaneous or surgical), depending on the presence of symptoms, extent, or clinical presentation of the disease (acute or chronic). Recently, new technologies have promoted substantial progress in the diagnosis and treatment of CAD. These technologies include the physiological assessment of CAD, which complements the findings of the anatomical evaluation; cardiac biomarkers; and microRNAs, which help in the detection of CAD. New drugs have allowed the reduction of cardiovascular events and advances in invasive treatment. Aided by robotics, shockwave, nanotechnology, stem cells, and three-dimensional printing can be useful to visualize the extent of the coronary occlusion and stenosis (Central Illustration). In this article, we summarize the literature on recent advances regarding therapeutic strategies and diagnostic methods for CAD.

## 2. Diagnoses and Evaluation of Coronary Artery Disease

“But there is a disorder of the breast marked with strong and peculiar symptoms, considerable for the kind of danger belonging to it (...). The seat of it and the sense of strangling and anxiety with which it is attended, may make it not improperly be called angina pectoris.” [2].

Since Heberden’s description in 1772 [2], our understanding of CAD has considerably expanded. Its characteristics, natural history, and pathological peculiarities have been scrutinized, and methods for a more thorough clinical evaluation have been constantly emerging. In 1958, the first selective coronary arteriogram was performed by Dr. F. Mason Sones, which has become a cornerstone in CAD evaluation [3]. Cumulative knowledge has set the foundation for a rapidly evolving field, and novel methods in the evaluation of CAD help physicians to more accurately evaluate the burden of the disease upon their patients.

CAD may be assessed via anatomical or functional methods. The first relies on observing the disease by detecting physical obstruction to blood flow. The key to anatomical methods is coronary angiography, which delineates coronary anatomy using contrast media and radiation. Upon similar premises, but with the advantage of being a noninvasive method, coronary computed tomography (CCT) is increasingly being used in broader scenarios. In addition to luminal evaluation, CCT offers insights into vascular structures beyond the degree of obstruction and allows the evaluation of the coronary artery calcium (CAC) score, an important prognostic tool with the capacity to reclassify patients during primary prevention. Intracoronary imaging methods have also been developed, and intravascular ultrasonography (IVUS) and optical coherence tomography (OCT) have been incorporated in clinical practice. More recently, plaque evaluation technology has been developed, and near-infrared spectroscopy (NIRS) is a novel technique in this area (Table 1).

Coronary angiography is inaccurate for assessing the function of coronary lesions; often, stenosis that is deemed severe on angiographic assessment does not restrict coronary blood flow at rest or with maximal dilatation. The physiological assessment of CAD allows complementing the findings of the anatomical evaluation. Probably the most widespread physiological assessment method is the exercise electrocardiogram (ECG). Resting ECG may provide clues regarding CAD, such as signs of past events or ischemic changes; however, in stable patients, such changes are unlikely to occur without a higher than basal metabolic demand, because the obstruction in this scenario is fixed. Imaging methods, such as stress echocardiography, single-photon emission computed tomography (SPECT), cardiac magnetic resonance (CMR), or positron emission tomography (PET), have emerged as more accurate means of evaluating ischemia. Despite being distinct ischemia induction techniques, all these methods have an ischemia detection accuracy of above 80% [4]. As such, SPECT and CMR stress-perfusion have become methods used almost worldwide for noninvasive functional evaluation, allowing the characterization of specific ischemic myocardial segments, especially for individuals with suspected CAD with intermediate risk probability. Invasive functional assessment has also been developed, and catheter-based methods such as fractional flow reserve (FFR), instant wave-free ratio (iFR), coronary flow reserve (CFR), and quantitative flow ratio (QFR) are being added to the arsenal of CAD evaluation tools. Additionally, noninvasive methods for plaque-specific functional analysis have been developed; FFR and other similar measures can be performed with the aid of CCT (Table 2).

Finally, biomarkers have been investigated to aid with CAD detection. Circulating microRNAs have been proposed as a potential target in this context. Additionally, perivascular fat attenuation has recently been studied and associated with local immune-inflammatory response activation, which is closely related to plaque vulnerability [5]. Below, some of the aforementioned methods are discussed in more detail.

### 2.1. Anatomical Methods

#### 2.1.1. Intravascular Ultrasonography (IVUS)

Intravascular ultrasonography (IVUS) has played an important role in recent interventional evolution. It enables not only the anatomical evaluation of lesion severity and plaque morphology but also better stent deployment and intrastent obstruction evaluation [8]. Additionally, IVUS is an invaluable tool in the evaluation of patients with myocardial infarction with nonobstructive coronary arteries (MINOCA).

IVUS technology is based on the acquisition of intravascular images with the aid of a transducer in the tip of a catheter during interventional coronary angiogram (ICA), generating cross-sectional images of the arteries. The IVUS waves travel through the blood and then sequentially throughout the artery layers: intima (which partially reflects these waves, generating a bright image), media (usually dark), and adventitia (also bright) [9]. The cross-sectional nature of the image allows for a better appreciation of the plaque anatomy compared with ICA alone. The presence and degree of calcification in a lesion of interest can also be assessed. IVUS is particularly useful for detecting dissections and eccentric plaques not seen on ICA, which can be mechanisms for MINOCA detected on angiogram. Several clinical studies have evaluated its usefulness in clinical practice [10,11,12]. The assessment of the minimal lumen area (MLA) by intravascular imaging could be a surrogate for ischemia evaluation [13,14]. Park at al. showed that in patients with isolated ostial and shaft intermediate left main coronary artery stenosis, an IVUS-derived MLA of ≤4.5 mm^2^ was a useful index of a fractional flow reserve (FFR) of ≤0.80 [14]. However, this number varies according to the location of the lesion, which limits its use in daily clinical practice.

The Providing Regional Observations to Study Predictors of Events in the Coronary Tree (PROSPECT) study evaluated the use of IVUS for predicting events after acute coronary syndrome (ACS) [15]. The 3-year cumulative rate of major adverse cardiovascular events (MACEs) was 20.4%. Almost half of these were in nonculprit lesions, and plaque-related risk factors were a plaque burden of 70% or greater (hazard ratio (HR) 5.03) and an MLA of 4 mm^2^ or less (HR 3.21). Additionally, the authors used radiofrequency analysis to estimate plaque composition, and those classified as thin-cap fibroatheromas had a higher risk as well (HR 3.35). Furthermore, IVUS-guided PCI has shown superiority in reducing MACEs [16] and target vessel failure [17].

#### 2.1.2. Near-Infrared Spectroscopy (NIRS)

NIRS imaging offers the ability to penetrate blood and tissue to detect lipid core-containing coronary plaques. The technology is based on the spectral analysis of the plaque with the aid of catheter-based equipment. The concept of spectroscopy is based on different materials having different interactions with light, which can basically be absorbed or scattered within the tissue [18,19]. These different interactions lead to the establishment of spectral “fingerprints”, enabling the observer to infer the composition of a determined object [20]. This technology has been widely used in other fields of science, allowing, for example, the identification of the composition of distant astronomical objects in astrophysics [21].

The electromagnetic spectrum is composed of an array of wavelengths, from longer to shorter: radiofrequency, microwave, infrared, visible light (and its respective colors), ultraviolet, X-rays, and gamma rays [22]. Whereas the eye can interpret radiation in the visible light part of the spectrum only, different equipment can evaluate other wavelengths [20]. This is where NIRS is valuable.

The NIRS technique has been evaluated in some trials [23,24]. The aforementioned PROSPECT trial assessed plaque composition with radiofrequency analysis, which is similar to NIRS [15]. The Providing Regional Observations to Study Predictors of Events in the Coronary Tree II (PROSPECT II) trial evaluated the ability of IVUS and NIRS to detect vulnerable plaques within the coronary arteries [25]. Patients in the acute/subacute phase of an MI (up to 4 weeks) were recruited. After successful treatment of all flow-limiting lesions in patients with recent MI, intravascular imaging was performed in the proximal 6–10 cm of all three coronary arteries with a combination NIRS–IVUS catheter. The authors found a higher risk of subsequent MACE in patients with identified highly lipid lesions (adjusted OR 2.27, 95% CI 1.25–4.13) and a high plaque burden (with additive effect). Those with lesions with both a high plaque burden and highly lipid lesions had a 7% 4-year rate of MACEs, whereas those with high plaque burden without signs of highly lipid content had a rate of 2.2%.

#### 2.1.3. Optical Coherence Tomography (OCT)

Optical coherence tomography (OCT) is another intravascular imaging method that relies on the reflection of light to form cross-sectional images. Compared with IVUS, it has both advantages and disadvantages.

The first and more notable advantage of OCT is its higher imaging resolution, which may lead to more accurate appreciation of thin-cap fibroatheroma, stent malapposition, coronary dissection, and neointimal proliferation, among others [24,25]. Additionally, OCT can be used to more thoroughly evaluate calcified plaques and thrombi, because IVUS evaluation can be limited in this specific scenario. [25,26,27]. Another advantage is that OCT allows for a better 3D modeling of the vessels, if desired [25,27].

However, OCT demands injection of a contrast medium to achieve blood clearance, as blood may disturb the light signal, and this may culminate in a larger quantity of contrast [26,27]. Another downside to the need for blood clearance is that lesions located at the ostia might not be adequately assessed, as achieving clearance in these locations is difficult. MLA may also be evaluated with OCT, which has lower numerical thresholds than IVUS and has a good positive predictive value (PPV) and a low negative predictive value (NPV) for significant FFR lesions [26,27].

### 2.2. Functional Methods

#### 2.2.1. Fractional Flow Reserve (FFR)

Fractional flow reserve (FFR) is an invasive index of the functional severity of stenosis determined from coronary pressure measurement during cardiac catheterization [28]. This technique is used to evaluate the maximum possible distal flow to the myocardium supplied by a stenotic artery as a fraction of the normal maximum flow. It relies on two pillars, the first being the positioning of a pressure-monitoring guide wire distal to the specific lesion and the second being the administration of a vasodilator to achieve maximal hyperemia. The pressure distal to the lesion is then compared with a normal reference, such as the aortic root pressure. Under normal circumstances, the ratio of a given point on an epicardial vessel to this normal reference should be one, meaning flow is not remarkably obstructed. Pijls et al. showed that an FFR < 0.75 reliably discriminates coronary stenosis, whether associated with inducible ischemia or not [29]. In another study, Pijls et al. compared FFR with multiple noninvasive tests (bicycle exercise testing, thallium scintigraphy, stress echocardiography with dobutamine, and quantitative coronary arteriography) [30]. All the patients with an FFR < 0.75 had at least one of these tests compatible with ischemia, which was reverted after angioplasty or surgery. This evidence strongly suggests that FFR is an accurate method for the detection of ischemia. However, the clinical implications of these findings remain uncertain.

The Fractional Flow Reserve Versus Angiography for Multivessel Evaluation (FAME) study was the first to assess the clinical impacts of FFR [31]. The results from this study indicated that FFR-guided PCI is associated with a significantly lower incidence of MACEs, defined as a composite of death, MI, or any repeat revascularization, compared with routine angiography-guided PCI in patients with multivessel coronary disease, without a significant increase in procedure time and with lower costs and resource use.

The Fractional Flow Reserve versus Angiography for Multivessel Evaluation 2 (FAME 2) study evaluated whether FFR-guided PCI plus the best available medical therapy would be superior to the best available medical therapy alone in reducing the MACEs among patients with stable CAD [32]. PCI was only considered for patients with an FFR ≤ 0.80. The enrollment was prematurely stopped owing to a highly significant difference in the incidence rates of the primary end point between the PCI and medical therapy groups. However, patients were not blinded to the presence of a lesion thought to be hemodynamically important, and a large majority of the events were revascularizations performed based on symptoms that mainly occurred in the early half of the trial.

The Fractional Flow Reserve versus Angiography for Multivessel Evaluation (FAME) 3 trial compared coronary artery bypass grafting (CABG) with FFR-guided PCI for three-vessel CAD [33]. The findings demonstrated that FFR-guided PCI using current-generation DES did not meet the criteria for noninferiority compared with CABG at one-year follow up (HR 1.50; 95% CI 1.10–2.20; p for noninferiority = 0.35). However, the short follow-up time (one year) may have underestimated an increased benefit of CABG, which, in other studies, showed a beneficial cumulative effect over several years of follow-up.

In summary, FFR appears to be a safe method to determine which lesions do not warrant treatment in addition to visual analysis. However, what it represents in terms of clinical value is still debated.

#### 2.2.2. Computed Tomography Fractional Flow Reserve (CCTA-FFR)

Despite some degree of controversy, FFR has been implemented in clinical practice for the evaluation of stable CAD. However, its invasive nature precludes its more widespread use.

Coronary computed tomography angiography (CCTA) is a noninvasive method that has been increasingly used in patients with chest pain, providing acceptable anatomical, but limited functional, evaluation. Upon these premises, CCTA-based FFR (CCTA-FFR) has emerged as an alternative to merge anatomical and functional evaluation through a noninvasive test. Whereas FFR is based on the direct measurement of distal and proximal pressures, CCTA-FFR is based on fluid dynamics principles and calculations [34].

Min et al. compared 252 patients who underwent CCTA, CCTA-FFR, ICA, and FFR regarding ischemia detection [35]. Compared with ICA and FFR, CT and CCTA-FFR were found to have 73% diagnostic accuracy, 90% sensitivity, 54% specificity, 67% PPV, and 84% NPV. The addition of CCTA-FFR seemed to increase the yield of CT alone, with a larger area under the curve (AUC) (0.81 vs. 0.68). Of note, this study described about 6 h of time required for each CCTA-FFR case. Additionally, 46.5% of patients had obstructive CAD on ICA, whereas 53.2% had this finding on CT. Of these, 37.1% had FFR < 0.80 and 53.3% had CCTA-FFR < 0.80. Similarly, the Diagnosis of Ischemia-Causing Stenosis Obtained via Noninvasive Fractional Flow Reserve (DISCOVER-FLOW) study compared CCTA-FFR with ICA and FFR and found a diagnostic accuracy of 84.3%, sensitivity of 87.9%, specificity of 82.2%, PPV of 73.9%, and NPV of 92.2% [36].

A second iteration of the CCTA-FFR algorithm was tested against invasive angiography with FFR in 251 patients with suspected CAD in the Analysis of Coronary Blood Flow Using CT Angiography: Next Steps (NXT) trial [37]. This trial reported that the diagnostic accuracy, sensitivity, specificity, PPV, and NPV for CCTA-FFR on a per-patient basis were 81%, 86%, 79%, 65%, and 93%, respectively.

In the Prospective Longitudinal Trial of FFRCT: Outcome and Resource Impacts (PLATFORM) trial, CCTA-FFR was incorporated into clinical practice, and patients with chest pain with planned noninvasive testing or ICA were first submitted to CCTA-FFR or received planned care [38]. Hence, four cohorts were built: (1) standard noninvasive testing; (2) ICA; (3) CCTA-FFR first for patients with planned noninvasive testing; (4) CCTA-FFR first for patients with planned ICA. When comparing these groups, 73.3% of patients in the ICA cohort had an angiogram without obstructive CAD, whereas only 12.4% of patients who underwent CCTA-FFR first and still needed ICA later had angiograms without obstructive CAD. MACEs (death, MI, or unplanned revascularization) were low throughout the cohorts and did not differ among strategies. The rates of revascularization were similar among groups. This finding suggests that deferring ICA based on CCTA-FFR is safe and may prevent patients from undergoing invasive procedures.

The Fractional Flow Reserve Derived from Computed Tomography Coronary Angiography in the Assessment and Management of Stable Chest Pain (FORECAST) trial showed that compared with routine management, the use of CCTA-FFR decreased the need for invasive angiography but did not reduce costs or MACEs [39].

In conclusion, CCTA-FFR apparently has a high accuracy when FFR is used as the gold standard, adding discriminative power to CT alone and reducing false-positive findings by associating a functional evaluation with this anatomical method.

#### 2.2.3. Instant Wave-Free Ratio (iFR)

After the clinical validation of FFR, new techniques have been developed in the search for a method that unites physiological assessment with simpler procedures. Instant wave-free ratio (iFR) is one of these methods that obviates the need for a hyperemic agent such as adenosine. The iFR is calculated by measuring the resting pressure gradient across a coronary lesion during diastole when microvascular resistance is low and stable [40,41].

A hyperemic agent in FFR is needed because the analysis of flow derives from a direct measure of pressure, and, for these variables to linearly correlate, coronary resistance must be low and constant. The authors who developed this technique found a period during diastole (when the myocardium (and, consequently, the microvasculature) is relaxed) when the coronary resistance is naturally low and constant [41]. While monitoring resistance, the wave-intensity analysis of this segment of time reflects a wave-free period, which means a more controlled environment to assess the functional value of a specific stenotic lesion. The measurement of the gradient during this period is similar to measurement with the infusion of a hyperemic agent such as adenosine [42].

Götberg et al. compared iFR with FFR in patients with stable CAD and ACS [40]. The iFR-guided revascularization strategy was noninferior to the FFR-guided revascularization strategy with respect to the rate of MACEs at 12 months. The FFR group had more significant lesions and more stents deployed. Additionally, the FFR group had a higher rate of chest discomfort during the procedure (68.3% vs. 3%), including moderate and severe chest pain. When using FFR as a reference standard, iFR achieved an accuracy of approximately 80% [42,43]. Nonetheless, iFR has potential advantages compared with FFR, such as independence from hyperemic medications (resulting in increased patient comfort) and reduced procedural time and costs.

#### 2.2.4. Coronary Flow Reserve (CFR)

Coronary flow reserve (CFR) is a ratio that corresponds to how much extra flow the coronary circulations can dispose of in situations when demanded, such as stress or vasodilation [44,45,46]. CFR is extremely useful in the diagnosis of microvascular CAD because visual assessment is impossible in such small vessels, leaving functional evaluation as the only choice for objective diagnoses. In addition to this distinct function, CFR predicts MACEs and all-cause mortality [44]. Clinical trials evaluating CFR-guided treatment are lacking, but some evidence shows that it may add prognostic data to FFR alone [46,47].

CFR may be invasively or noninvasively measured with the aid of positron emission tomography (PET-CT), cardiac magnetic resonance (CMR), or even stress echocardiography. Regarding noninvasive assessments, the first method is PET-CT, in which a radiotracer (usually rubidium-82 or nitrogen-13-ammonia) is used to acquire dynamic images (for instance, at rest and with a vasodilator) and evaluate myocardial blood flow at rest and at hyperemia, from which the CFR is then calculated [48]. CMR evaluates CFR by measuring flow at rest and peak stress at the coronary sinus [49]. To measure CFR with stress echocardiography, in addition to evaluating wall motion abnormalities, flowmetry in the mid-distal left anterior descending (LAD) artery is evaluated at basal and peak stress with dipyridamole [50].

#### 2.2.5. Quantitative Flow Ratio (QFR)

The quantitative flow ratio (QFR) is a novel semiautomated method: a novel functional evaluation technique that can be rapidly calculated during ICA using two projections and computer software, obviating the need for adenosine-induced hyperemia and the insertion of pressure-wires. Basically, the software creates a three-dimensional model from the two ICA projections and uses fluid dynamics to evaluate flow by counting the frames necessary for the contrast to enter and leave the segment of interest [51,52].

This promising physiological assessment was clinically tested against FFR in the Wire-Free Functional Imaging II (WIFI II) study [53]. Compared with FFR as the gold standard, a QFR ≤0.80 cutoff achieved an AUC of 0.86 (95% CI, 0.81–0.91), a sensitivity of 77%, a specificity of 86%, a PPV of 87%, and an NPV of 75%. A hybrid QFR–FFR approach enabled wire-free and adenosine-free procedures in 68% of the cases.

The Functional Lesion Assessment of Intermediate Stenosis to Guide Revascularization (DEFINE-FLAIR) [54] and the Instantaneous Wave-free Ratio versus Fractional Flow Reserve in Patients with Stable Angina Pectoris or Acute Coronary Syndrome (iFR-SWEDEHEART) [40] trials have also demonstrated that iFR is equivalent to FFR in terms of incidence of MACEs in patients experiencing angina or MI. The studies have also showed that iFR resulted in markedly less patient discomfort and reduced procedure-related adverse events compared with FFR.

The Concordance Between FFR and iFR for the Assessment of Intermediate Lesions in the Left Main Coronary Artery: A Prospective Validation of a Default Value for iFR (iLITRO-EPIC07) study showed that concordance between FFR and iFR in patients with intermediate left main coronary artery (LMCA) stenosis was moderate (80%). Furthermore, in case of discordance, IVUS tended to be more similar to FFR in classifying stenosis significance [55]. Thus, an approach based on a combination of IVUS and physiology for intermediate LMCA lesions appears to define whether revascularization can be safely deferred.

#### 2.2.6. Index of Microcirculatory Resistance (IMR)

The index of microcirculatory resistance (IMR) was first developed by Fearon et al. and is calculated from estimates of maximal distal coronary flow during hyperemia and pressure [56]. Ng et al. showed that IMR is superior to CRF for assessing the coronary microcirculation by being more reproducible and less hemodynamically dependent than CFR [57] because it is not dependent on resting values. Moreover, IMR is not affected by epicardial stenosis severity [58]. IMR ≥ 25 units indicates abnormal microcirculatory function.

#### 2.2.7. Hyperemic Microvascular Resistance (HMR)

HMR is defined as the ratio between the distal coronary pressure (Pd) and maximal coronary flow velocity during hyperemia; an HMR > 1.9 mmHg/cm/s is diagnostic of microcirculatory dysfunction [59]. Williams et al. showed that HMR has higher diagnostic accuracy than IMR in predicting coronary flow reserve (AUC 0.82 vs. 0.58, *p* < 0.001; sensitivity and specificity 77% and 77% vs. 51% and 71%, respectively) and myocardial perfusion reserve index (AUC 0.85 vs. 0.72, *p* = 0.19; sensitivity and specificity 82% and 80% vs. 64% and 75%, respectively) [60]. As with IMR, HMR is based on the application of Ohm’s law (resistance = pressure/flow), only substituting thermodilution-derived volumetric flow with Doppler-derived flow velocity [61]. Table 3 summarizes the main differences between the methods of invasive assessment of coronary microcirculation.

Other invasive methods of coronary microvascular function assessment include minimal microvascular resistance (MMR) and resistive reserve ratio (RRR), which use Doppler and thermodilution principles, respectively.

De Waard et al. showed that MMR allows the evaluation of microcirculatory dysfunction, irrespective of epicardial flow [62]. For this reason, MMR is proposed as a clinical measure of microvascular disease in both ischemic and nonischemic cardiopathy. Moreover, Lee et al. showed that the RRR has incremental prognostic implications in patients with CAD and elective PCI [63].

#### 2.2.8. Novel Resting Nonhyperemic Pressure Ratios

More recently, several wire-based nonhyperemic pressure ratios (NHPRs) in addition to iFR have been developed. These novel indices measure the ratio of distal coronary artery pressure (Pd) to aortic pressure (Pa) but differ in the phase of the cardiac cycle used for measurement (Table 4). Essentially, the diagnostic accuracy of these novel NHPRs is almost identical to that of iFR. In addition, retrospective studies have shown comparable prognostic performance between NHPR and iFR [64,65]. These findings suggest that novel NHPRs and iFR could be clinically applied in similar manners.

For all variables cited above, using a physiological metric as a dichotomous pathway to decide on revascularization is probably incorrect.

#### 2.2.9. Cardiac Magnetic Resonance Novelties

CMR is a widely used diagnostic modality for detailed investigation of structural and functional cardiac pathologies. The higher spatial resolution of CMR allows stress-perfusion phase identification of myocardial ischemia, even if small ischemic areas are involved, with accuracy of 94% [4]. CMR allows reclassifying suspected CAD patients to a more appropriate risk category of death and MI [66], and it is a key diagnostic tool in the evaluation of patients presenting with myocardial infarction with nonobstructive coronary arteries (MINOCA).

Within the recent development of quantitative CMR techniques, T1 mapping, which evaluates intracellular edema and extracellular matrix expansion, can precisely identify MI without contrast injection by native T1 phase changes [67].

### 2.3. Biomarkers

#### 2.3.1. Pericoronary Fat Attenuation Index (FAI)

Identifying markers of plaque vulnerability might be useful to improve coronary risk stratification even in secondary prevention patients. Pericoronary fat assessment may help identify vulnerable plaques associated with increased risk of cardiovascular events [53] by differentiating the lipid content in the perivascular tissue surrounding the vessels with and without inflammatory activity.

Antonopoulos et al. developed a method to evaluate the fat variability with computerized tomography (CT), the pericoronary fat attenuation index (FAI) [68]. The FAI is an average of the tissue attenuation, where the higher the lipid content and the larger the adipocyte size, the lesser the attenuation found and the more negative the FAI. This index correlates well with lipid content in histologic assessments, local inflammatory markers, and vessel inflammation detected by 18F-fluorodeoxyglucose uptake assessed by PET-CT. In a post hoc analysis of two cohorts of the Cardiovascular RISk Prediction using Computed Tomography (CRISP-CT) study, perivascular FAI improved risk prediction in comparison with regular procedures [69]. In this analysis, the FAI values around the right coronary artery (RCA) and the left anterior descending artery (LAD) predicted all-cause and cardiac mortality, which led the authors to suggest that using the RCA perivascular FAI can act as a surrogate for global coronary inflammation. The optimal cutoff suggested by the authors is a perivascular FAI ≥ −70.1 Hounsfield units (HU) (the perivascular fat attenuation spectrum extends from around −190 HU in the adipose to −30 HU in the aqueous phase), which led to an HR 5.62 for cardiac mortality and an HR 3.69 for all-cause mortality in the validation cohort.

Sun et al. showed that increased pericoronary FAI values on coronary computed tomography angiography were associated with vulnerable plate components in patients with non-ST elevation acute coronary syndrome (ACS) [5].

#### 2.3.2. MicroRNAs

MicroRNAs (miRNA) are small sequences of nucleotides (18–25) that do not generate protein but regulate many biologic processes by gene silencing [70]. The roles of their up- or downregulation in the development of diseases have been a growing area of interest. Many miRNAs related to cardiovascular disease, including left ventricular hypertrophy, ischemic heart disease, heart failure, hypertension, and arrhythmias, have been identified [71,72,73].

Different profiles of miRNA expression have been noted in patients with and without CAD. For instance, different sets of miRNA have been identified among normal vascular smooth muscle cells and myofibroblasts, which developed in the plaque-generating process. Other miRNAs have been found to correlate with atherosclerotic lesions in the endothelium, inflammation, and lipid metabolism, all of which play an important role in CAD [72]. High-throughput sequencing, quantitative real-time polymerase chain reaction (RT-qPCR), and microarrays are the major quantification methods that are currently being used [70,74].

In the ACS scenario, Li et al. showed that the blood levels of miRNA-1 correlated to conventional troponin T levels with similar, but not superior, accuracy (AUC 0.85, 95% CI 0.81–0.89) [75]. Other biomarkers with high accuracy for MI are miRNA-208a and miRNA-499 [76,77].

MiRNAs may either serve as the cause/facilitators of disease or as byproducts of the disease process. Ideally, identifying specific miRNAs in a patient might lead to early diagnoses or even better risk prediction. However, many unanswered questions remain, and obtaining a better understanding as to how miRNAs distribute through different ethnicities, ages, and genders; determining how to uniformly acquire and evaluate the biological samples; and cost-effectively operationalizing this technology are crucial for the development of this promising area.

#### 2.3.3. Polygenic Risk Scores for CAD

Cardiogenomics is emerging as a revolution in cardiology. In the field of prevention, the detection of polymorphisms associated with increased risk of CAD has been a target for better risk assessment. Current risk scores consider variables such as age, sex, blood pressure, and cholesterol, which strongly impact the final result. As age is an important risk factor for CAD, younger patients might have low 10-year risk but a high lifetime risk, with an underestimation of this risk. Polygenic risk scores (PRS) might help with understanding a patient’s lifetime risk and complement the risk evaluation in these patients. These scores were developed from genome-wide association studies, which considered databases with genetic variants that were tested for associations with specific phenotypes, such as CAD [78]. These studies require expertise and can be statistically complex due to the problem of multiple comparisons, but these scores constitute a field with a promising future.

Many genetic variants have already been identified as risk-enhancing, and most of them do not seem to increase risks by augmenting known risk factors, such as low-density lipoprotein (LDL) cholesterol or blood pressure. PRS has been analyzed in the populations of the Further Cardiovascular Outcomes Research with PCSK9 Inhibition in Subjects with Elevated Risk (FOURIER) [79] and Evaluation of Cardiovascular Outcomes After an Acute Coronary Syndrome During Treatment with Alirocumab (ODYSSEY OUTCOMES) [80] trials of proprotein convertase subtilisin/kexin type 9 inhibitors (PCSK9i). In the nested cohort study of 14,298 unrelated European-ancestry patients enrolled in the FOURIER trial, a PRS consisting of 27 single-nucleotide polymorphisms was applied to stratify patients in quintiles to evaluate their genetic risk [81]. Clinical risk was evaluated by means of categorical variables such as high LDL, hypertension, smoking, and diabetes mellitus. In this analysis, higher scores on the PRS were associated with higher rates of clinical events. This study suggested that adding genetic risk evaluation can lead to improved patient selection for PCSK9 inhibition, as the relative and absolute risk reductions (RRR and ARR, respectively) in the events were 13% and 1.4%, respectively, for high clinical risk alone and 31% and 4%, respectively, for high genetic risk (quintile five), irrespective of clinical risk. Post hoc analyses of the ODYSSEY OUTCOMES trial [82], a PRS comprising almost 7 million genetic variants, were performed regarding the risk of MACEs and treatment efficacy among different genetic risk strata. Patients in the placebo group that were above the 90th percentile in this PRS had a higher risk of MACEs than patients under the 90th percentile (17% vs. 11.4%). In the treatment efficacy analysis, alirocumab (a PCSK9i) produced a larger reduction in risk in patients above the 90th percentile (6% of ARR and 37% of RRR) compared with those in the lower-risk strata under the 90th percentile (1.5% of ARR and 13% of RRR).

Despite being a rapidly growing area of research, prospective data to recommend such scores as routine practice are insufficient. Moreover, these emerging biomarkers still should be compared with proven available prognostic laboratory cardiovascular biomarkers such as troponin and high-sensitivity C-reactive protein (hsCRP) in larger studies to determine their diagnostic and prognostic role.

## 3. Novelties in Drug Treatment of Coronary Artery Disease (CAD)

The primary goals of CAD treatment are to alleviate symptoms and prevent complications in patients with stable CAD and to improve blood flow and restore heart function as quickly and as best as possible in patients with ACS [83,84]. Traditionally, this is accomplished through optimal medical therapy and the consideration of myocardial revascularization with either PCI or coronary artery bypass grafting (CABG). The optimal medical therapy consists of beta blockers, calcium channel blockers, nitrates, angiotensin-converting enzyme (ACE) inhibitors, and statins for stable CAD patients, and thrombolytics and antiplatelets agents for ACS patients.

Recently, novel therapies for CAD have been evaluated in randomized clinical trials, some of which are still ongoing. In this section, we focus our discussion on these promising therapies only.

### 3.1. Reverse Cholesterol Transport

Reverse cholesterol transport is a term used to describe the efflux of excess cellular cholesterol from peripheral tissues and its return to the liver for excretion in the bile [82]. Enhancing foam cell cholesterol efflux by high-density lipoprotein cholesterol (HDL-C) particles is the first step of reverse cholesterol transport, which is a promising antiatherogenic strategy [85,86].

To date, agents that increase HDL-C levels, such as niacin and cholesterol ester transfer protein inhibitors, have not been proven to reduce cardiovascular events, but their impact on HDL-C efflux capacity is variable, complex, and possibly influenced by adjunctive statin therapy [87].

### 3.2. Vascular Endothelial Growth Factor

Vascular endothelial growth factor (VEGF) is a dimeric glycoprotein that mediates the formation of new blood vessels during angiogenesis by activating the VEGF receptor. It may play a role in the progression of human coronary atherosclerosis as well as in the recanalization processes in obstructive coronary diseases [88].

The ongoing epicardial delivery of XC001 gene therapy for refractory angina coronary treatment (EXACT) clinical trial is evaluating whether XC001, a novel adenoviral vector expressing multiple isoforms of vascular endothelial growth factor (VEGF), promotes an enhanced local angiogenic effect in patients with refractory angina [89].

### 3.3. Selatogrel

The use of a P2Y12 inhibitor as a component of dual antiplatelet therapy in patients with an acute coronary syndrome (ACS) is well established.

Selatogrel, a novel, potent, reversible, and selective 2-phenylprimdine-4-carboxamide that is subcutaneously administered is under development. The results from preclinical, phase 1, and phase 2 trials have confirmed that the agent provides sustained and reversible P2Y12 platelet inhibition with an acceptable safety profile and with a larger therapeutic window compared with the oral P2Y12 inhibitors [90,91].

In patients with MI, the administration of a single dose of 8 or 16 mg of selatogrel was safe and induced a profound, rapid, and dose-related antiplatelet response [92]. In patients with chronic CAD, selatogrel provided prompt, potent, and consistent platelet P2Y12 inhibition sustained for ≥8 h, with reversible effects within 24 h [93].

The ongoing Selatogrel Outcome Study in Suspected Acute Myocardial Infarction (SOS-AMI) clinical trial will evaluate the clinical efficacy of selatogrel in patients with acute MI (NCT04957719).

### 3.4. Revacept

Revacept is an intravenous competitive antagonist to platelet glycoprotein VI (GPVI) that efficiently binds to collagen and effectively mimics the GPVI pathway, blocking platelet aggregation.

The results of animal models have shown that revacept is effective in preventing platelet adhesion and thrombus formation in arterial lesions induced in the carotid artery without affecting bleeding time [94,95,96] and in reducing neointimal hyperplasia and the levels of markers of cell proliferation and macrophage infiltration, being able to serve, in the latter case, as a therapeutic agent for PCI and stent implantation [97]. Further preclinical investigation showed that revacept strongly inhibits human plaque-induced thrombosis in ex vivo superfusion models using human patient blood and plaques gained during carotid surgery [98].

In a phase II clinical trial, revacept reduced the combined safety and efficacy endpoint (any stroke or death, transient ischemic attack, MI, coronary intervention, and bleeding complications) in patients with symptomatic internal carotid artery after 11.2 ± 2.3 months of follow-up [99]. However, the Intracoronary Stenting and Antithrombotic Regimen: Lesion Platelet Adhesion as Selective Target of Endovenous Revacept in Patients With Chronic Coronary Syndromes Undergoing Percutaneous Coronary Intervention (ISAR-PLASTER) phase 2 trial showed that the addition of revacept to currently recommended antithrombotic therapy in the setting of PCI in patients with stable ischemic heart disease did not reduce myocardial injury [100]. New clinical trials are needed to evaluate the role of revacept in reducing the occurrence of major cardiovascular events.

### 3.5. Inclisiran

Inclisiran is a small interfering ribonucleic acid (siRNA) that prevents hepatic PCSK9 production [101].

Ray et al. showed that inclisiran reduced PCSK9 and LDL-C levels among patients at high cardiovascular risk that had elevated LDL-C levels [102]. Furthermore, an analysis of 1561 and 1617 patients from the ORION-10 and ORION-11 trials, respectively, found that ~50% reductions in LDL-C levels were obtained with inclisiran, which was subcutaneously administered every 6 months [103]. The long-term treatment with inclisiran provided sustained reductions in LDL-C and PCSK9 concentrations and was well tolerated over 4 years [90].

The ongoing randomized trial assessing the effects of inclisiran on clinical outcomes among people with cardiovascular disease (ORION-4) trial will provide more robust evidence of both the efficacy and safety of inclisiran in terms of MACEs (NCT03705234).

### 3.6. AZD5718

The 5-lipooxygenase activating protein (FLAP) is essential for the production of leukotrienes through the 5-LO pathway. The inhibition of this pathway was hypothesized to reduce mortality, morbidity, and cardiovascular hospitalization in patients with CAD by slowing the progression of atherosclerosis, to enhance coronary microvascular function, and to improve ventricular contractility following MI [104].

AZD5718 is a novel FLAP antagonist that acts at the first step of biosynthesis to block production of all leukotrienes [105]. The ongoing AZD5718 Phase IIa Study to Evaluate Efficacy, Safety and Tolerability of Oral AZD5718 in Patients with Coronary Artery Disease (FLAVOUR) clinical trial will evaluate the efficacy and safety of AZD5718 in patients with MI [106].

### 3.7. Rivaroxaban

Rivaroxaban is a factor Xa inhibitor anticoagulant that inhibits thrombin formation and plays a pivotal role in both coagulation and platelet activation.

The Anti-Xa Therapy to Lower Cardiovascular Events in Addition to Standard Therapy in Subjects with Acute Coronary Syndrome-Thrombolysis in Myocardial Infarction 51 (ATLAS ACS 2-TIMI 51) trial compared rivaroxaban at a low dose of 2.5 mg b.i.d. with a placebo in stabilized patients treated predominantly with aspirin and clopidogrel following ACS. They found a reduction in composite of MI, stroke, or cardiovascular death at the expense of increased bleeding with rivaroxaban [107].

The Cardiovascular Outcomes for People Using Anticoagulation Strategies (COMPASS) trial randomized 27,395 patients with stable CAD or peripheral arterial disease (PAD) to rivaroxaban 2.5 mg b.i.d. plus aspirin, rivaroxaban 5 mg b.i.d. alone, or aspirin alone [108]. Compared with those assigned to random aspirin alone, patients assigned to rivaroxaban plus aspirin showed a significant 22% decrease in cardiovascular mortality and a 49% decrease in ischemic stroke. Those assigned to combination therapy had a significant 70% increase in major bleeding events, with the gastrointestinal tract being the most common site of major bleeding.

The results of post hoc secondary analysis of the Atrial Fibrillation and Ischemic Events with Rivaroxaban in Patients with Stable Coronary Artery Disease (AFIRE) trial with 2215 participants showed that rivaroxaban monotherapy was associated with lower risks of total thrombotic and/or bleeding events in patients with atrial fibrillation and stable CAD compared with rivaroxaban and antiplatelet therapy [109].

### 3.8. Colchicine

Evidence is increasingly showing that inflammation plays a key role in the pathogenesis of atherosclerosis. The research aimed at the improvement of outcomes in patients with CAD that has recently shown a promising role of colchicine, a drug with anti-inflammatory properties [110,111,112]. Following this anti-inflammatory theory, some traditional drugs with this action were tested. Nonsteroidal anti-inflammatory drugs were associated with an increased risk of MI [113], whereas corticosteroids have been found ineffective or associated with an increased risk of cardiac rupture [114,115].

The Low-Dose Colchicine (LoDoCo) trial (532 patients) showed that colchicine (0.5 mg daily), added to standard care, reduced cardiovascular events in patients with stable CAD [116].

The low-dose colchicine (LoDoCo) 2 trial (5522 patients) showed that colchicine (0.5 mg, once daily) reduced the risk of cardiovascular events in patients with chronic CAD [117]. An exploratory LoDoCo2 trial showed that continued long-term anti-inflammatory therapy with low-dose colchicine produced a consistent reduction in major cardiovascular events year-by-year during 5 years of follow-up [118].

The Colchicine Cardiovascular Outcomes Trial (COLCOT) study also showed a benefit in ischemic outcomes with the use of colchicine in patients with recent MI [119]. The findings of the COLCOT study are complementary to those of the LoDoCo 2 trial. However, the Colchicine in Patients with Acute Coronary Syndrome (COPS) trial showed no effect of the use of colchicine in patients with ACS on cardiovascular outcomes compared with the use of a placebo (6.1% vs. 9.5%, *p* = 0.09) [120].

Despite these conflicting results, colchicine appears to be promising in the prevention of cardiovascular events in patients with CAD, either in chronic or acute settings, mainly due to the acceptable safety profile and low cost [121]. The inhibition of inflammation will likely become the fourth cornerstone of CAD treatment, together with the lowering of LDL-C, inhibition of platelet aggregation and control of additional risk factors.

### 3.9. Sodium-Glucose Cotransporter 2 (SGLT-2) Inhibitors

A new generation of cardiorenal protective agents, SGLT2 inhibitors, has been studied in patients with type 2 diabetes, heart failure (HF), and nephropathy [121,122,123,124,125,126,127,128,129,130]. These trials have focused on outpatients with and without concomitant CAD and have demonstrated consistent efficacy for the prevention of hospitalization for HF and a reduction in worsening kidney disease and inconsistent reduction in atherosclerotic outcomes or cardiovascular death [122,123,124,125,126,127,128,129,130,131]. However, a gap exists in the literature regarding the use of these drugs in CAD, either in acute or chronic settings [132].

The EMpagliflozin in acute MYocardial infarction (EMMY) trial showed that empagliflozin was associated with a significantly larger reduction in NT-proBNP over 26 weeks, which was accompanied by a significant improvement in functional and structural echocardiographic parameters in patients with MI [133].

The ongoing Empagliflozin in Patients Postmyocardial Infarction (EMPACT-MI) trial will provide more information on the safety and efficacy of empagliflozin compared with a placebo in patients hospitalized for MI with or at high risk of new onset HF, in addition to standard care [134].

### 3.10. PCSK9 Inhibitors

PCSK9 is an enzyme that binds to the epidermal growth factor of domain A of the low-density lipoprotein receptor (LDL-R), inducing the degradation of this receptor. These reduced LDL-R levels result in decreased LDL-C metabolism, which can lead to hypercholesterolemia. The inhibition of PCSK9 function by monoclonal antibodies was found to be an effective method to reduce cholesterol levels [135]. In combination with high-intensity or maximum tolerated statins, alirocumab and evolocumab reduced LDL-C by 46–73% compared with a placebo and by 30% compared with ezetimibe [81,82]. Moreover, they resulted in a large reduction in future cardiovascular events. When statins are not tolerated or cannot be prescribed, PCSK9 inhibition reduced LDL-C levels when administered in combination with ezetimibe [136].

A current reanalysis of the FOURIER trial compared mortality data in the primary results publication with those in the Clinical Study Report (CSR). After readjudication, deaths of cardiac origin were numerically higher in the evolocumab group than in the placebo group, suggesting possible cardiac harm. The trial was terminated early when a nonsignificantly higher risk of cardiovascular mortality was observed with evolocumab, which was numerically larger in this readjudication [137].

## 4. Invasive Treatment Novelties

Despite substantial recent improvement in the invasive treatment of coronary heart disease, especially coronary stent structural evolution, novel technologies might provide additional endothelium and myocardial healing as well as reduce cardiovascular events for specific coronary lesions. Additionally, professional welfare is an important concern, with new tools available to assist with medical workers’ well-being and to lessen labor hazards. In this section, we describe the role of new technologies in the management of CAD.

### 4.1. Robot-Assisted Percutaneous Coronary Intervention

Although recent technological improvement in PCI has enabled the treatment of complex coronary lesions with a lower burden due to complications such as stent restenosis and thrombosis, interventional professionals’ occupational radiation and orthopedics hazards are of concern [138,139]. As such, remote operational cardiovascular devices systems have recently been developed to reduce such labor exposure hazards and potentially improve the stent-length selection [140]. The CorPath 200 system is the most used model. It is composed of a bedside unit (articulated arm, robotic drive, and devices), a remote cockpit with radiation shields and central control unity of console, hemodynamic monitors, and X-ray foot pedals. However, arterial access, diagnostic angiography, and the guiding catheter must be performed manually.

The safety and feasibility of CorPath Robot-PCI was evaluated in prospective nonrandomized trials [141,142]. The Percutaneous Robotically Enhanced Coronary Intervention (PRECISE) trial enrolled a total of 164 patients with at least 50% stenosis in a coronary artery that could be treated with a single stent at both elective and urgent PCI [143]. Device technical success was achieved in 98.8%; no deaths, strokes, Q-wave MI, or revascularization occurred in the 30 days after the procedures. The radiation exposure for the primary operator was 95.2% lower than that with the traditional table position. However, the coronary lesions in this study were mainly noncomplex. Therefore, in the Complex Robotically Assisted Percutaneous Coronary Intervention (CORA-PCI) trial, 334 PCI procedures were performed on 315 patients (20% with ACS) presenting with a higher lesion complexity (78.3% type B2/C on robot-PCI (R-PCI) group vs. 68.3% on manual PCI) [144]. Technical success with R-PCI was 91.7%, and comparable clinical outcomes, without adverse effects on stent use or fluoroscopy time, were observed between the groups.

Future technological improvement in R-PCI might open new perspectives regarding the feasibility and safety of fully remotely stenting patients in a separate physical location using a combination of robotics and telecommunication [145,146].

### 4.2. Shockwave Coronary Intravascular Lithotripsy System

The PCI of severe calcified coronary (CAC) lesions might be challenging because heavy coronary calcification is a risk factor for coronary dissection, vessel perforation, MI, stent restenosis, and thrombosis [147,148,149,150]. In addition, it is associated with stent underexpansion, malapposition, and structural damage [147,148,149,150]. Atheroablative technologies available for CAC PCI lesions can be limited to guidewire issues, and they may promote slow flow, no reflow, and coronary dissection [151,152,153,154,155,156,157]. Here, a novel technology based on renal calculi treatment, denominated the shockwave intravascular lithotripsy (S-IVL) system, was developed. Pulsatile mechanical shockwaves are delivered by a balloon catheter, analogous to contemporary balloon catheters, that disrupt calcium with soft tissue sparage [157,158]. The safety and efficacy of this new technique have been demonstrated in prospective nonrandomized studies [159,160,161,162] that evaluated stable and acute CAD patients with events caused by heavily calcified coronary lesions submitted to S-IVL prior to PCI. The pooled analysis of these four studies that enrolled 628 patients showed a 92.4% procedural success with a low rate of MACEs (cardiac death, all MI, and target vessel revascularization) of 7.3% [163]. Prior MI, bifurcation, and long-length lesions were independent predictors of MACEs and a lack of procedural success. Any post-IVL angiographic complication occurred in only 2.1% of procedures. Thus, S-IVL is a promising and safe technology for treating severe calcified lesions.

### 4.3. Stem Cell Therapy for Ischemic Heart Disease

Stem cells are capable of self-renewal and differentiation; thus, they might be beneficial for cardiac regeneration after MI and heart failure. The several kinds of stem cells include embryonic stem cells (derived from blastocysts), mesenchymal nonhematological stromal cells from bone marrow, and induced pluripotent stem cells (iPS) derived from any tissue and genetically changed to act as an embryonic cell [164,165]. Although stem cell therapy demonstrated myocardial recovery in rodents after MI [166], the clinical human benefits of this therapy are still under debate.

A Cochrane review of bone marrow stem cells application in patients after acute MI did not show improvement in mortality, life quality, or left ventricular function [167]. However, another small study that evaluated the intracoronary infusion of autologous cardiosphere-derived cells found myocardium recovery of infarcted area and lessened scar tissue [168]. Stem cell survival after transplantation is brief; thus, their therapeutic effects might be most related to the paracrine propriety of secretion of cytokines and growth factors of several antiapoptosis, angiogenesis, inflammation, and cell-recruitment signaling pathways [169,170,171,172,173,174,175]. As such, the injection of small vesicles from endocytic origin, called exomes, which are secreted by stem cells, that contain proteins, RNA, and miRNA has potential cardioprotective effects similar to those of cellular stem treatment but with longer stability, with low immune response, and without tumoral risk [176]. Some prelusive studies have suggested exomes treatment improves ventricular function after myocardial damage [177,178].

Future research and technology development on stem cells’ preconditioning, the route of administration, exomes, and tissue bioengineering are promising in the coming decades to improve myocardial recovery after acute ischemic events in stable CAD patients as well as to decrease heart failure burden.

### 4.4. Nanotechnology

Nanoparticle (NP) properties enable direct delivery of drugs to targets with higher drug circulation time and solubility; thus, NPs may increase drug efficacy with lower doses and lesser side effects [179,180,181]. This technology has the potential to treat coronary heart disease, reducing stent restenosis and targeting atheroma plaques. Smart systems of NPs carried by stents may provide sustained local drug delivery at a stenting site, being a promising therapy for this specific treatment [180]. Several encapsulating NP systems have been developed to deliver antirestenosis drugs such as liposomes [182], micelles [179], and polymeric NPs [177,180]. Additionally, the use of gel-like NPs loaded with rapamycin in a carotid-injured rat model demonstrated endothelium healing and decreased hyperplasia [183]. Gene therapy is another evolving strategy for vessel wall healing and restenosis inhibition [184,185,186].

In a prospective preclinical study with animal models using distinct nanocoating systems composed of hyaluronic acid and plasmid DNA, nanobiohybrid hydrogel carrying endosomolytic Tat peptide together with DNA produced re-endothelization [187,188]. Nanotechnology was also applied to produce a nanotexture polymeric coating to regulate drug release from stents. For example, in vitro studies of polylactic-co-glycolic acid (PLGA) NPS, a biocompatible polymer, demonstrated controlled liberation of paclitaxel without disruption [189]. Several direct atherosclerotic pathways can be addressed by NPs at atheroma plaques [178].

From animal models, researchers have reported inflammation attenuation via the NPs delivery of interleukin-10 (IL-10) [190]; NPs combined with small interfering RNA (siRNA) inhibited leukocyte adhesion [191]; antiangiogenic NP anti-integrins combined with local statin avoided plaque neovascularization [192,193,194]. Macrophage blocking [192,193] and local thrombolytic and anticoagulant delivery by NPs [195,196,197,198,199,200,201,202] have shown promising results regarding plaque stabilization.

### 4.5. Three-Dimensional Printing

Three-dimensional (3D) printing, first introduced in 1986 [203], is a technology that allows physical 3D objects to be manufactured from a digital geometrical model [204,205]. Three-dimensional printing has many educational and clinical applications: it might be useful for medical instruction and teaching as well as to improve communication with patients [206,207].

For CAD, 3D models provide a detailed visual assessment of the coronary arterial bed, which might be important for evaluating stenotic regions, to prepare to interventional procedures [208,209,210], to perform invasive exams simulations [211], and, possibly, to test patient coronary-bed-specific stenting [204]. In addition, 3D printing combined with other imaging methods enables simulations of invasive diagnostic measures to test and set standard references of evolving diagnostic tools against the in vitro gold standard [212]. Future 3D printing development might provide individual patient-based device innovations.

## 5. Conclusions

Despite advances in pharmacological and revascularization techniques decreasing mortality, CAD remains one of the main causes of morbidity and mortality in the world. Since the early 2010s, some therapeutic and diagnostic strategies have improved the understanding and treatment of the disease. The identification of potential therapeutic and diagnostic targets is necessary to develop novel treatment and diagnostic strategies for CAD. However, robust clinical evidence is still scarce. Future research in this domain should be focused on stem cell therapies, new angiogenic treatment modalities, and new diagnostic modalities.

## Figures and Tables

**Table 1:** Characteristics of methods for anatomical assessment of coronary artery disease. Abbreviations: IVUS: intravascular ultrasonography; OCT: optical coherence tomography; NIRS: near-infrared spectroscopy; PCI: percutaneous coronary intervention; NA: not applicable. Adapted from Maehara et al. [6].

	IVUS	OCT	NIRS
Resolution	+	++	NA
PCI (stent sizing, expansion)	++	++	NA
Necrotic core	+	+	++
Detection of thin cap	±	++	-
Thrombus	±	+	-
Stent tissue coverage	+	++	-
Calcification	++	-	-
Remodeling	++	-	-
Neointimal hyperplasia	+	++	-
Requires blood-free field of view	No	Yes	No

**Table 2:** Characteristics of methods for physiological assessment of coronary artery disease. Abbreviations: CCTA: coronary computed tomography angiography; CFR: coronary flow reserve; FFR: fractional flow reserve; HMR: hyperemic microvascular resistance; iFR: instantaneous wave-free ratio; IMR: index of microcirculatory resistance; QFR: quantitative flow ratio. Adapted from Maitre-Ballesteros et al. [7].

Method	Advantages	Limitations
FFR	Prognostic studies available	Guidewire: cost; complication Hyperemia: cost; side effect of adenosine Increase time of procedure
iFR	Validated by noninferiority studies vs. FFR Hyperemia-independent Quicker than FFR	Guidewire: cost; complication Hyperemia: cost; side effects of adenosine Specific software required
QFR	Hyperemia-independent No pressure wire	Specific software required Precise acquisition of angiography Manual correction by expert
CCTA-FFR	Noninvasive Increase performance of CCTA	Cost Offline analysis
CFR	Study all coronary tree Prognostic performance Overall assessment (macro- and microcirculation)	Variability: intrinsic plus variable resting condition Guidewire: cost; complication Hyperemia: cost; side effects of adenosine Increase time of the procedure
IMR	Microcirculatory analysis	Guidewire: cost; complication Hyperemia: cost; side effect of adenosine Increase time of the procedure
HMR	Microcirculation analysis	Guidewire: cost; complication Hyperemia: cost; side effect of adenosine Increase time of the procedure Doppler: additional cost; Doppler signal not analyzable in 30% of patients No cutoff value

**Table 3:** Invasive hemodynamic parameters for assessing coronary microcirculation. Abbreviations: HMR: hyperemic microvascular resistance; IMR: index of microcirculatory resistance; Pd: distal mean coronary pressure; T: mean transit time; V: coronary flow velocity.

	IMR	HMR
Method	Thermodilution	Doppler
Definition	T_hyperemia_ × P_d_	P_d_/V_hyperemia_
Abnormal value	≥25 units	>1.9 mmHg/cm/s
Limitations	T: surrogate of flow Requires hyperemia	V: surrogate of flow Requires hyperemia

**Table 4:** Characteristics of novel resting nonhyperemic pressure ratios. Abbreviations: DFR: diastolic hyperemia-free ratio; dPR: diastolic pressure ratio; FFR: fractional flow reserve; iFR: instantaneous wave-free ratio; NHPR: nonhyperemic pressure ratio; Pa: aortic pressure; Pd: distal coronary artery pressure; RFR: resting full-cycle ratio.

Pressure Index	Hyperemia	Cutoff	Calculation of Index
FFR	Required	≤0.80	Average Pd/Pa during entire cycle at hyperemia (typically averaged over 3 beats)
Resting Pd/Pa	NHPR	≤0.91	Average Pd/Pa during entire cycle at hyperemia (typically averaged over 3 beats)
RFR	NHPR	≤0.89	Instant lowest filtered Pd/Pa ratio during the entire cardiac cycle (over 5 beats)
iFR	NHPR	≤0.89	Average Pd/Pa during wave-free period (over 5 beats)
DFR	NHPR	≤0.89	Average Pd/Pa during period between Pa < mean Pa and down-sloping Pa (over 5 beats)
dPR	NHPR	≤0.89	Average Pd/Pa during entire diastole (over 5 beats)

## Data Availability

Not applicable.
